# Post-Activation Performance Enhancement of Lower Limb with Variable Resistance Back Squat at Different Depths

**DOI:** 10.3390/jfmk10030347

**Published:** 2025-09-11

**Authors:** Choon Yeow Ng, Danny Lum

**Affiliations:** 1 Singapore Weightlifting Federation, Singapore 469643, Singapore; 2 High Performance Sport Institute, Singapore 397630, Singapore; 3 Sport Performance, and Nutrition Research Group, School of Allied Health, Human Services, & Sport, La Trobe University, Melbourne, VIC 3086, Australia

**Keywords:** countermovement jump, countermovement depth, time to take-off, mean propulsion force

## Abstract

Background: This study compared the acute effect of performing quarter (QS) and parallel (PS) squat with variable resistance (VR) on countermovement jump (CMJ) performance. Methods: Fifteen resistance trained athletes (age: 27.0 ± 3.7 years, bodyweight: 68.4 ± 7.7 kg, height: 169.7 ± 6.9 cm) performed either the QS or PS to induce post-activation performance enhancement on two separate occasions. During each session, participants performed three repetitions of baseline CMJ prior to performing three repetitions of either QS or PS at their three-repetition maximum load. Participants then performed the post-test CMJ after a five-minute recovery period. Results: Both conditions resulted in increased jump height (QS: *p* < 0.001, *g* = 0.19, PS: *p* < 0.001, *g* = 0.35). Countermovement depth and time to take-off were significantly decreased in QS (*p* < 0.001, *g* = 0.63 and *p* = 0.005, *g* = 0.30, respectively) but significantly increased in PS (*p* = 0.027, *g* = 0.39 and *p* < 0.001, *g* = 0.36, respectively). Mean propulsion force was significantly increased in QS (*p* < 0.001, *g* = 0.23) but significantly decreased in PS (*p* = 0.083, *g* = 0.13). PS resulted in greater change in jump height (*p* < 0.001, *g* = 1.34) and time to take-off (*p* = 0.005, *g* = 1.25), while QS resulted in greater change in countermovement depth (*p* < 0.001, *g* = 2.33) and mean propulsion force (*p* < 0.001, *g* = 1.67). Conclusions: The results showed that performing PS and QS with VR was effective in enhancing CMJ height. However, the two conditions resulted in participants adopting different jump strategies when performing the post-test CMJ.

## 1. Introduction

Post-activation performance enhancement (PAPE) is a phenomenon where the ability to perform various sports related task is improved after including high intensity activity during the warm up process [1,2]. Researchers have investigated various methods to attained PAPE including lifting heavy weights, performing plyometric and ballistic exercises with variable resistance, and performing maximal voluntary isometric contraction [2,3,4,5].

As the countermovement jump (CMJ) is an important movement skill in many sports [6], multiple studies have employed various PAPE activities to enhance CMJ performance [2,5]. In particular, the back squat exercise is one of the most commonly used PAPE activity due to its similar biomechanical movement pattern [7]. Previous studies have compared the effects of PAPE on lower limbs with back squat using free weights [5,7,8,9,10,11,12]. According to a study by Esformes and Bampouras [8], performing parallel back squat (PS), which involved squatting to a depth where the upper thigh was parallel to the ground, was found to be more effective than quarter squats (QSs), which involved squatting a knee angle of 135°, in inducing PAPE. It was explained that the deeper depth achieved from PS induced a greater gluteal activation, thus having a greater effect on lower limb force generation ability. Indeed, Caterisano et al. [13] reported that the activation of gluteus maximus increased progressively as squat depth increased. However, in one meta-analysis review, Seitz and Haff [5] stated that there was a greater PAPE effect after shallow squats in stronger and weaker individuals. It was also noted that squat depths which were parallel and below would induce greater levels of acute fatigue. With these contrasting views, it is necessary to further investigate the most optimal squat depth for inducing PAPE to enhance CMJ performance.

Free weights, also known as constant resistance, provide a consistent load throughout a range of motion (ROM) [14]. However, it may not be the most optimal method for muscle activation as constant resistance does not consider for the change in lever arm and angle with the association to the torque relationship especially when lifting heavy loads [15]. When that happens, it is often termed as the “sticking region”, which refers to the region from the initial maximum velocity to the first local minimum velocity of the free weight (barbell) [16,17]. It has been reported that muscle activity differs during pre-sticking, sticking and post-sticking region [17,18], which led to the suggestion that potentiation of contractile elements is reduced at the range of movement beyond the sticking region. To overcome this limitation, variable resistance (VR) can be introduced in addition to free weights to reduce the mechanical disadvantage experienced when using free weights [19].

Variable resistance comes in the form of elastic bands or chains changes in resistance intensity as individuals perform a given exercise at different range of movement [19,20]. The inclusion of VR during physical activities have been reported to result in increased muscle activation and greater physical performance than performing free weights [21,22,23]. For example, greater activation of quadricep muscles was observed when VR was included in a squat compared to free weights [21]. In addition, both Masel et al. [22] and Mina et al. [23] reported greater improvement in jump performance after performing squat that included VR than free weights alone.

To date, no study has compared the PAPE effects of QS and PS that include VR. In addition, previous studies investigating the effects of QS and PS on jump performance did not measure important jump metrics such as time to take-off and countermovement depth that are factors affecting jump height [24]. Therefore, the purpose of the current study was to compare the PAPE effects of QS and PS with the addition of VR on CMJ performance. Based on knowledge of previous findings using back squats with free weights, it was hypothesised that PS would result in greater PAPE effect.

## 2. Material and Methods

### 2.1. Participants

The sample size was determined using G*Power version 3.1.9.2 (Dusseldorf, NRW, Germany), a statistical power of 0.8, a significance level of 0.05, and an effect size of approximately 0.5 based on previous studies evaluating the immediate impact of PAPE activity on jump performance [25]. The analysis indicated that the minimum required sample size for this study was 10 participants. Fifteen resistance trained national athletes (10 males—age: 27.7 (3.6) years, height: 172.6 (6.1) cm, body weight: 72.5 (5.1) kg; 5 females—age: 25.6 (4.0) years, height: 163.8 (4.5) cm, body weight: 60.2 (4.8) kg) from badminton (*n* = 5), cycling (*n* = 3), diving (*n* = 3) and track sprinting (*n* = 4) participated in this study. The inclusion criteria for the study were (1) 18–35 years of age; (2) more than one year of resistance training experience; (3) able to back squat more than 1.5 times body weight; and (4) not sustaining any musculoskeletal injury or chronic illness. Participants were informed to abstain from caffeine and alcohol for at least 24 h prior to each session. Habitual coffee drinkers were allowed to continue with their usual daily dose of coffee. All testing commenced after obtaining approval from the Institutional Review Board of the Singapore Sport Institute (SC-Full-016, approved on 9 October 2018). Written consent was sought prior to participation.

### 2.2. Testing Procedure

**Figure 1:**
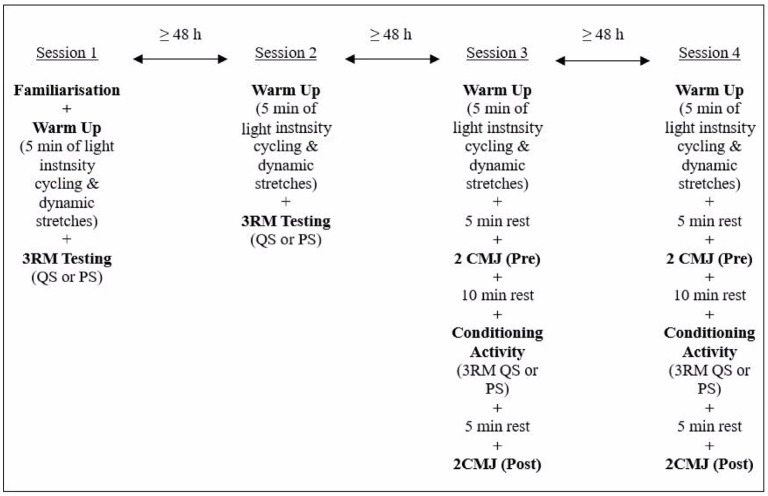
Experimental timeline.

This study utilised a within participant counterbalance approach to establish the optimum squat depth that would induce PAPE when performing squats with variable resistance. Participants attended four separate sessions (Figure 1), which included one familiarisation with a testing session, one testing session and two experimental sessions. During the familiarisation session, participants were briefed on the experimental procedures. During the first and second sessions, participants under-went three-repetition maximum (3RM) tests for QS and PS, one in each session. During the subsequent two testing sessions, the PAPE effects of QS and PS were measured. The order of QS and PS was randomised.

Upon arrival at the testing venue for each session, participants were required to perform a standard warm up protocol, which included 5 min of self-paced cycling on a cycling ergometer followed by 10 repetitions of body weight squat, hip hinge, forward lunge, submaximal CMJ and ankle hops. Participants were provided with a 1 min recovery period prior to performing the main activity of each session.

#### 2.2.1. 3 Repetition Maximal Test

**Figure 2:**
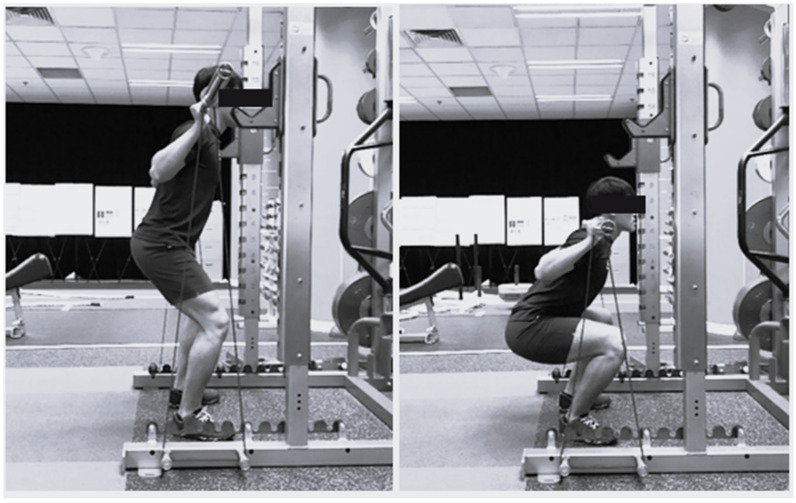
Quarter (**left**) and parallel (**right**) squat with VR attached.

The 3RM testing procedure follows that described by Esformes and Bampouras [8]. Participants attempted 3 repetitions of a load. Upon a successful attempt, the load was increased by 2.5–5 kg for the subsequent attempt. Participants were given a 5 min recovery period between trials. The 3RM was determine within 5 trials. For the PS, participants were required to descend until the inguinal fold was lower than the patella (Figure 2). For the QS, participants were required to descend until a ~135° knee joint angle was reached. Upon completion of the 3RM test on the second session, the measurement of the resistance contributed by VR was measured. With the participant standing on a force plate (ForceDecks, VALD Performance, FD4000, Queensland, Australia) with sampling at 1000 Hz with an unloaded barbell, resistance bands were attached until the amount of load from the bands reached ~20% of 3RM for both QS and PS at the standing position [26].

#### 2.2.2. Countermovement Jump

The CMJ was performed on the same force plate with participants keeping their arms akimbo to prevent any arm swing. Participants were required to perform three attempts during both baseline and post-PAPE activity measures, with each attempt separated by 30 s. The commercially available ForceDecks software version 2.1.1 (VALD Performance, ForceDecks, Queensland, Australia) was used to analyse and generate the jump variables including jump height, time to take-off, mean propulsion force and countermovement depth. The best of the three attempts was used for further analysis. The software used a 20 N offset from the measured body mass, measured before the jump, to define the initiation of the jump; therefore, participants were asked to stand as still as possible for >1 s prior to the commencement of the countermovement [27].

### 2.3. Postactivation Performance Enhancement Activity

For the PAPE activity, participants performed 1 set of 3RM (with 20% of the load from resistance band) (Sanctband, Perak, Malyasia) of either QS or PS in a randomized and counterbalanced fashion. Before executing the 3RM load, participants were instructed to perform progressive warm ups of the squat starting with an unloaded barbell with the specified intensity of variable resistance attached. Subsequent increments were included the load and repetitions of 60% 3RM × 5 repetitions, 75% 3RM × 3 repetitions and 90% 3RM × 1 repetition. The final set of 3RM × 3 repetitions was used as the PAPE activity. Each warm up set was separated by 2 min. Participants were provided a 5 min recovery period before they performed the post-conditioning activity CMJ attempts [8]. The current study did not include a control condition where no PAPE activity was performed as this study replicated the protocol of previous study [8] with only the change in the type of resistance used.

### 2.4. Statistical Methods

All data were analysed using JASP 0.19.3. Within session test–retest reliability was assessed using two-way, mixed intraclass correlation coefficients (ICCs) and coefficient of variation (%CV) for all measured variables. ICC values were deemed as poor, moderate, good, or excellent if the lower bound 95% confidence interval (CI) of ICC values were <0.50, 0.50–0.74, 0.75–0.90, or >0.90, respectively [28]. Acceptable within-session variability was classified as <10% [29].

Analysis of all variables was conducted to ensure normality of data and all assumptions of the statistical test were met. A factorial repeated measures analysis of variance (ANOVA) was conducted using 2 × 2 (condition × time) to analyse pre- and post-squat CMJ within and between each condition (QS and PS). All significant interaction effects found in the analyses were further analysed using pairwise correction (Sidak) to correct for type I errors. Significance was set at *p* < 0.05 with a 95% confidence interval and all data were presented as mean (SD).

Hedge’s *g* effect size was used to compare the difference between force variables obtained between testing devices. Hedge’s *g* was computed as follows: (i) trivial effect size if *g* < 0.25; (ii) small effect size *g =* 0.25–0.50; (iii) moderate effect size if *g =* 0.51–1.0; (iv) large effect size if *g* > 1.0 [30].

## 3. Results

### 3.1. Reliability

The reliability data are displayed in Table 1. Excellent reliability was observed for jump height and countermovement depth. Good reliability was observed for mean propulsion force, and moderate reliability was observed for time to take-off.

### 3.2. Jump Height

Significant time (*p* < 0.001) and time × group (*p* = 0.002) interaction was observed for jump height with no group effect observed (Table 2). Jump height was significantly increased after both QS (*p* < 0.001, 95%CI = 1.5 to 2.6, *g* = 0.19) and PS (*p* < 0.001, 95%CI = 2.8 to 4.3, *g* = 0.35). PS resulted in a greater change in jump height than quarter condition (*p* < 0.001, 95%CI = −5.2 to −1.9, *g* = 1.34).

### 3.3. Time to Take-Off

While significant time × group interaction (*p* < 0.001) was observed for time to take-off, there was no significant time or group effect (Table 2). Time to take-off was significantly decreased in QS (*p* = 0.005, 95%CI = −0.051 to −0.011, *g* = 0.30), but significantly increased in PS (*p* = 0.027, 95%CI = 0.006 to 0.080, *g* = 0.39). PS resulted in greater change in time to take-off than QS (*p* = 0.005, 95%CI = −18.6 to −4.0, *g* = 1.25).

### 3.4. Mean Propulsion Force

There was significant time × group interaction (*p* = 0.001) for mean propulsion force. But no significant time or group effect was observed (Table 2). QS resulted in significant increase in mean propulsion force (*p* < 0.001, 95%CI = 42.8 to 94.2, *g* = 0.23), while PS resulted in a non-significant decrease in mean propulsion force (*p* = 0.083, 95%CI = −80.5 to 5.6, *g* = 0.13). QS resulted in a greater change in time to mean propulsion force than parallel condition (*p* < 0.001, 95%CI = 3.9 to 9.3, *g* = 1.67).

### 3.5. Countermovement Depth

Significant time (*p* = 0.011) and time × group (*p* < 0.001) interaction was observed for countermovement depth with no group effect observed (Table 2). Countermovement depth was significantly decreased in QS (*p* < 0.001, 95%CI = −2.3 to −0.8, *g* = 0.63), but significantly increased in PS (*p* < 0.001, 95%CI = 2.2 to 5.3, *g* = 0.36). PS resulted in greater change in countermovement depth than QS (*p* < 0.001, 95%CI = −22.7 to −11.4, *g* = 2.33).

## 4. Discussion

The purpose of the current study was to compare the PAPE effects of QS and PS with the addition of VR on CMJ performance. Results of the study showed a significant increase in CMJ performance after both conditions with greater improvement observed in PS. The changes in jump height after both conditions were due to different mechanisms. While QS resulted in a significant decrease in time to take-off and countermovement depth and increase in mean propulsion force, PS resulted in the exact opposite direction for the change in these variables. Nevertheless, the current results supported the hypothesis that a greater PAPE effect would be observed in PS.

Previous studies conducted on PS at similar intensities (85–90% 1RM × 3 reps) also found PS to be effective in improving jump performance [8,9,10,31]. Esformes and Bampouras [8] attributed their findings to the increased activation of the gluteus maximus from a deeper squat which resulted in more torque for hip extension. However, in the present study, the load experienced by the participants varies throughout the range of motion due to the use of VR. This resulted in a reduction in effective load around the “sticking point” during the concentric phase of the squat but a gradual increase in load while returning to the upright position during PS [21]. This occurrence would have been the same for QS. The increased muscle activation throughout the full range of movement during the QS in the present study due to the addition of VR could have been the reason for the PAPE effect observed which did not occur in the study by Esformes and Bampouras [8]. Nevertheless, the current findings also supported that of Esformes and Bampouras’s study [8] in showing that PS resulted in greater improvement in jump height.

The novelty of the current study was the inclusion of the analysis of the CMJ time to take-off, mean propulsion force and countermovement depth. While multiple studies have reported acute increases in jump height after performing heavy squats [8,9,10], none of these studies have included other CMJ variables to explain the kinetic and kinematic changes that accompanied the change in jump height. The current results showed that the depth of the squat performed during the PAPE activity influenced the countermovement depth of the subsequent CMJ attempts. Specifically, QS and PS resulted in a decrease and an increase in countermovement depth, respectively, during the post-conditioning activity CMJ. The change in countermovement depth provides insights for the change in both time to take-off and mean propulsion force. The increase in countermovement depth would have led to more time taken for the centre of mass to travel the specific distance as observed in PS. On the other hand, the decrease in countermovement depth would then result in a decrease in time to take-off as observed in QS.

The increase in countermovement depth was reported to increase jump height [6,32]. The increase in countermovement depth allowed for a greater amount of time for the participants to generate a higher amount of impulse, which eventually led to a greater jump height, hence, the greater improvement in jump height observed in PS. However, despite the decrease in countermovement depth and time to take-off, QS also resulted in a significant increase in jump height. This was likely because of the greater mean propulsion force generated in QS which led to an increase in resultant impulse. This observation suggests that there was an increase in the rate of force development after performing QS, which allowed participants to develop a higher amount of force within a shorter period of time. Esformes and Bampouras [8] did not observe a PAPE effect after QS possibly due to the greater fatigue induced from lifting a heavier absolute load in QS as compared to PS. However, in the current study, VR was included instead of just free weights alone. Seitz et al. [33] reported that the inclusion of VR required only a minute of recovery between conditioning activity and criterion measure to induce PAPE effects. This recovery time was less than that required for free-weight conditioning activities [2,5]. Hence, this indicates that the inclusion of VR either induces a higher magnitude of PAPE than fatigue, or simply lower levels of fatigue than conditioning activities involving only free weights.

Several limitations should be considered when interpreting the current findings. Firstly, while it has been reported that inducing PAPE with VR may require less recovery time, the current study adopted the recovery duration commonly used when performing heavy resistance conditioning activities. Hence, it is unknown if a shorter recovery period would yield similar results. Secondly, participants in the current study were national athletes who perform a high volume of sports and resistance training regularly. Hence, they should be more fatigue-resistant than the general population or individuals with a lower training status and the current findings may not be applied to other populations. Thirdly, due to the current sample size, it was not possible to investigate the difference in response to the two PAPE protocols between athletes whose sport requires them to adopt a lower stance (e.g., speed skaters) as compared to those who stay in an extended position most of the time (e.g., runners). Finally, lifting velocity was not measured. Hence, the daily variation in physical condition was not assessed. However, the lack of significant difference in baseline CMJ height between conditions suggested that athletes’ physical condition was likely to be similar between testing sessions.

## 5. Conclusions

In conclusion, the present results showed that practitioners may adopt both QS and PS with VR to induce PAPE for the lower limbs during strength training for their athletes. However, practitioners may need to take the demands of the athletes’ sports into consideration on whether to adopt QS or PS, especially when the purpose is to induce PAPE during competition. For example, QS resulted in improved jump height and will also reduce the countermovement depth and time to take-off. This method may be more favourable for athletes in sports that require them to produce a high amount of propulsion force within a short period of time, such as track sprinting. On the other hand, PS resulted in a greater countermovement depth and time to take-off, which would be more suitable for athletes in sports that require them to generate high impulse and are less time constrained, such as weightlifting. In addition, if the purpose is to induce PAPE during training, practitioners may want to consider the training phase that the athletes are at and the main objective of the training. For example, PS may be more suitable to be paired with plyometric exercises that require longer contact time, which would enable the athletes to train their fast dynamic strength. On the other hand, QS may be more suitable to be paired with plyometric exercises that have shorter contact time, which would enable the athletes to train their reactive strength.

## Figures and Tables

**Table 1:** Reliability data of countermovement jump variables.

Variables	ICC (95%CI)	%CV (95%CI)
Jump height (cm)	0.995 (0.986–0.998)	1.3 (0.9–2.3)
Time to take-off (s)	0.871 (0.667–0.954)	2.9 (2.1–5.0)
Mean propulsion force (N)	0.949 (0.859–0.982)	2.7 (2.0–4.7)
Countermovement depth (cm)	0.985 (0.957–0.995)	3.3 (2.4–5.7)

CI = confidence interval, %CV = coefficient of variation; ICC = intra-class correlation.

**Table 2:** Pre- and post-conditioning activity measures for all countermovement jump variables.

Variables	Quarter				Parallel				Time	Group	Time × Group
	Pre	Post	%∆	*g*	Pre	Post	%∆	*g*			
Jump height (cm)	44.3 (9.8)	46.3 (10.2) **	4.6 (2.5) ^$$^	0.19	43.9 (9.7)	47.5 (10.5) ^##^	8.1 (2.6)	0.35	*p* < 0.001	*p* = 0.913	*p* = 0.002
Time to take-off (s)	0.687 (0.106)	0.656 (0.094) **	−4.2 (5.4) ^$$^	0.30	0.647 (0.103)	0.690 (0.111) ^#^	7.1 (11.2)	0.39	*p* = 0.548	*p* = 0.942	*p* < 0.001
Mean propulsion force (N)	1529.0 (279.0)	1597.5 (295.8) **	4.5 (2.9) ^$$^	0.23	1569.0 (298.1)	1531.7 (280.3)	−2.1 (4.6)	0.13	*p* = 0.194	*p* = 0.904	*p* = 0.001
Countermovement depth (cm)	33.3 (9.3)	31.7 (8.6) **	−4.5 (3.4) ^$$^	0.63	32.4 (9.9)	36.1 (10.2) ^##^	12.6 (9.5)	0.36	*p* = 0.011	*p* = 0.611	*p* < 0.001

** Denotes significant difference from Quarter Pre (*p* < 0.01); ^#^ Denotes significant difference from Parallel Pre (*p* < 0.05); ^##^ Denotes significant difference from Parallel Pre (*p* < 0.01); ^$$^ Denotes significant difference from Parallel %∆ (*p* < 0.01).

## Data Availability

Data will be available upon request.

## Abbreviations

The following abbreviations are used in this manuscript:

3RM	3 repetition maximum
CMJ	Countermovement jump
PS	Parallel squat
QR	Quarter squat
VR	Variable resistance
